# Secondary Hypertension Due to Underlying Takayasu Arteritis

**DOI:** 10.7759/cureus.28263

**Published:** 2022-08-22

**Authors:** Ahmad Raja, Summia Matin Afridi, Wen Wang, Ranjeet Kumar, Bahar Sumbul-Yuksel, Amrat Kumar

**Affiliations:** 1 Internal Medicine, Bassett Medical Center, Cooperstown, USA; 2 Internal Medicine, Ocala Regional Medical Center, Ocala, USA; 3 Internal Medicine, Hartford Hospital, Hartford, USA; 4 Rheumatology, AdventHealth Orlando, Orlando, USA

**Keywords:** autoimmune, uncontrolled hypertension, hypertension in young patients, secondary hypertension, takayasu arteritis

## Abstract

A young female patient in her early 20s of Hispanic descent presented to the hospital with new-onset chest pain and uncontrolled hypertension. She was found to have blood pressure in the 200s/100s. She was evaluated for causes of secondary hypertension and underwent computed tomography angiography (CTA) of her abdomen to rule out fibromuscular dysplasia, which showed abnormal thickening of lower thoracic and abdominal aorta extending into both renal arteries causing stenosis. This finding led to further investigations, and she was found to have elevated erythrocyte sedimentation rate (ESR) and C-reactive protein (CRP). Magnetic resonance angiography (MRA) was done, which confirmed the findings of periaortitis in the vessels as described above. A diagnosis of Takayasu arteritis (TA) was made, and the patient was treated with high-dose steroids with significant improvement in her symptoms.

## Introduction

Takayasu arteritis (TA) is a chronic vasculitis of unknown cause that most commonly affects the aorta and its branches. It is common in young people of Asian descent and usually presents with low-grade fever, weight loss, limb claudication, and weak or absent pulses. In our case, the patient presented with uncontrolled hypertension and chest pain unresponsive to treatment, which led to further testing that revealed inflammation of the aorta extending up to the renal arteries causing renal artery stenosis (RAS). Given the patient’s age, distribution of the aortitis, and elevated inflammatory markers, a diagnosis of Takayasu arteritis was made.

## Case presentation

A young female patient in her early 20s of Hispanic descent with a past medical history of migraine presented with new-onset chest pain. The patient’s chest pain was central, sharp, and non-radiating with no aggravating or relieving factors. She denied any history of smoking, alcohol, or drug use. She was recently admitted with similar complaints and was discharged home on amlodipine after a workup for acute coronary syndrome was normal. On further questioning, she did endorse around 20 pounds of unintentional weight loss in the past year. She denied claudication or any neurological symptoms. On physical examination, she was found to have elevated blood pressure around 200s/100s, equal in both arms, and sinus tachycardia up to 110, and she did not show typical signs of diminished pulses or carotid or aortic bruit.

**Figure 1:**
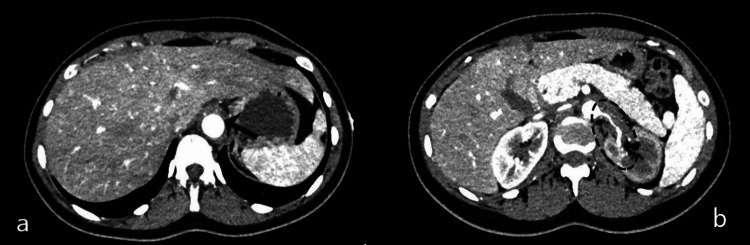
(a) Axial post-contrast CT angiogram of the abdomen showing mural wall thickening of the aorta (black solid arrow). (b) Axial post-contrast CT angiogram showing significant stenosis of the left renal artery (black dashed ellipse). CT: computed tomography

**Figure 2:**
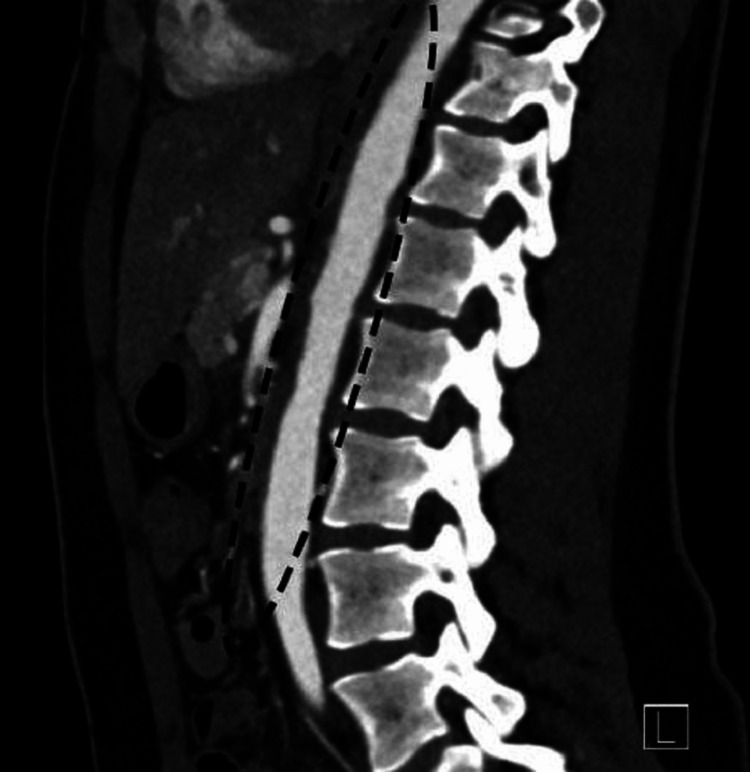
Sagittal CT angiogram of the abdomen showing an undulating appearance of the descending and abdominal aorta (black dashed ellipse). CT: computed tomography

**Figure 3:**
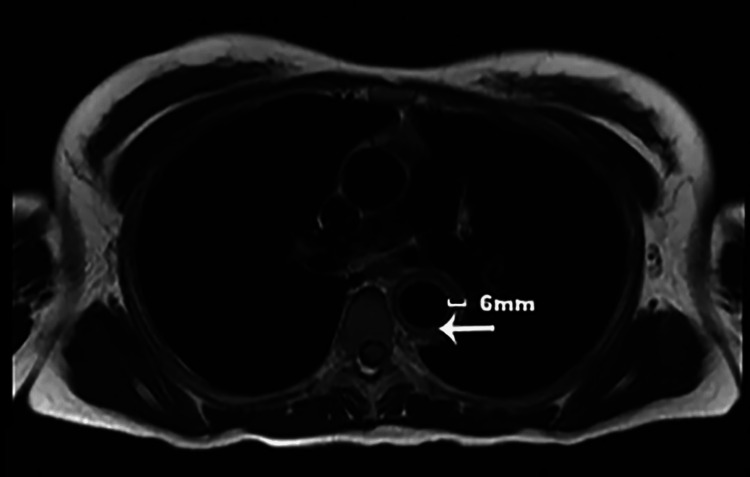
Axial double inversion recovery sequence of MRA of the chest showing the mural thickness of the descending aorta measuring 6 mm (white arrow). MRA: magnetic resonance angiography

**Figure 4:**
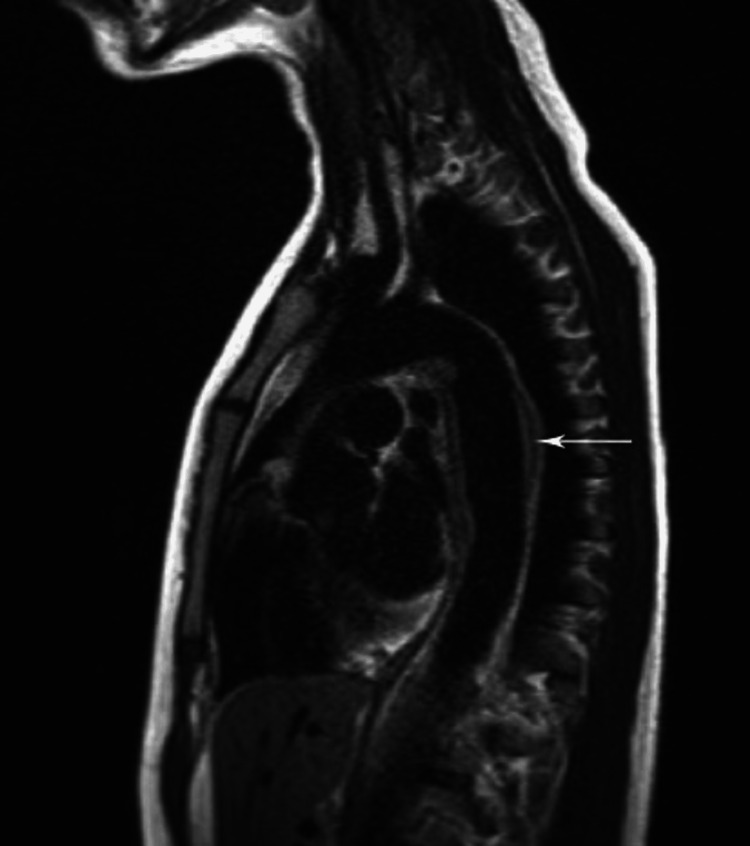
Sagittal double inversion recovery sequence of MRA showing mural thickening of the descending aorta without significant narrowing (white arrow). MRA: magnetic resonance angiography

The initial laboratory work revealed mild leukocytosis with a WBC count of 11.47 × 10^3^/uL and microcytic anemia with hemoglobin of 11.1 g/dL. Her C-reactive protein (CRP) was elevated at 51.1 mg/L, and her erythrocyte sedimentation rate (ESR) was >130 mm/hour. Electrocardiogram revealed sinus tachycardia. Her troponin level was normal. Her serum-free metanephrines and normetanephrines were normal. TSH and serum total calcium were within normal limits. She initially underwent renal ultrasound, which showed normal-sized kidneys and no signs of urinary tract obstruction. Due to high clinical suspicion of renal artery stenosis (RAS), computed tomography angiography (CTA) of the abdomen was done to rule out fibromuscular dysplasia, which showed abnormal findings consistent with thickening of lower thoracic and abdominal aorta extending into both renal arteries causing stenosis (Figure 1 and Figure 2). Magnetic resonance angiography (MRA) of the head, neck, chest, abdomen, and pelvis was ordered later, which showed periaortitis of the lower thoracic and abdominal aorta extending into both renal arteries (Figure 3 and Figure 4).

Given her young age and uncontrolled hypertension, the patient underwent an initial workup to rule out the causes of secondary hypertension. The differentials included pheochromocytoma, Cushing’s disease, secondary hyperaldosteronism, and fibromuscular dysplasia. She did not have any features or laboratory findings concerning for Cushing’s disease, and her workup for pheochromocytoma was negative. She underwent further imaging, which did show renal artery stenosis, but the cause identified was inflammatory, not fibromuscular dysplasia. Given her young age, history of weight loss, radiographic findings, and elevated inflammatory markers, the diagnosis of Takayasu arteritis was made.

The patient was transferred to the intensive care unit for close monitoring and was started on high-dose intravenous glucocorticoids. Her blood pressure was initially managed with nicardipine infusion and was later transitioned to an oral regimen of carvedilol, lisinopril, and hydrochlorothiazide. Rheumatology and vascular surgery services were consulted, and endovascular treatment was withheld due to the increased risk of complications given significant inflammation around the aorta. After the improvement in her symptoms and blood pressure, intravenous methylprednisolone was changed to oral prednisone taper.

The patient’s symptoms significantly improved after she was started on glucocorticoids. She was later discharged on oral prednisone and methotrexate. She was advised to follow up with rheumatology as an outpatient.

## Discussion

The majority of patients diagnosed with hypertension have essential hypertension; however, around 10%-15% of patients may have secondary hypertension [1]. The underlying cause of secondary hypertension differs with age. In young patients, coarctation of the aorta and intrinsic renal causes are more common, whereas, in adult patients, hyperaldosteronism, Cushing’s disease, pheochromocytoma, thyroid disease, obstructive sleep apnea, renal artery stenosis, and medications (e.g., contraception) are more frequently seen [2]. Obstructive sleep apnea was found to be the most common cause of secondary hypertension associated with resistant hypertension. In one study, out of 125 patients, 64% of patients with resistant hypertension had underlying obstructive sleep apnea, 5.6% with primary hyperaldosteronism, 2.4% with renal artery stenosis, 1.6% with intrinsic renal disease, 1.6% with oral contraceptive use, and 0.8% with underlying thyroid disorders [3]. Secondary causes of hypertension should be considered in patients with certain signs and symptoms including resistant hypertension, young age of onset, malignant hypertension, and hypertension associated with electrolyte derangements [1].

In addition to the abovementioned signs and symptoms, there are a few other findings that can help in identifying renovascular disease as an underlying cause of secondary hypertension, for example, 30% acute rise of serum creatinine after the use of angiotensin-converting enzyme (ACE) inhibitors or angiotensin receptor blockers (ARBs), asymmetric kidney size, flash pulmonary edema, unilateral abdominal bruit, and severe hypertension presenting after 55 years of age.

Renal artery stenosis (RAS) is defined as the narrowing of the renal arteries, which is reported to be around 1%-10% of the 50 million people in the United States. Atherosclerosis and fibromuscular dysplasia are the two most common causes of RAS. Other etiologies include arterial dissection, aortic aneurysm, thromboembolic disease, and vasculitis. Around 60% of the patients in India and the Far East were found to have renal artery involvement as the initial presentation of Takayasu arteritis [4].

Takayasu arteritis is an inflammatory vasculitis that involves medium- to large-sized arteries. It usually affects the aorta and its branches, including subclavian, pulmonary, iliac, and renal arteries [5]. It is usually seen in young females of Asian descent, but a few cases in Hispanic patients have also been described [6]. The patient can present with symptoms of limb claudication, angina, and neurological symptoms such as numbness, vertigo, and seizures. On physical examination, they can have absent or diminished pulses, carotid or aortic bruit, and difference in blood pressure in both arms [5]. Takayasu arteritis can be classified depending on the involved site (Table 1).

**Table 1:** Types of Takayasu arteritis.

Type	Artery involved
Type I	Supra-aortic involvement only
Type IIa	Aortic arch or ascending aorta with or without its branches
Type IIb	Involvement of the thoracic descending aorta, ascending aorta, aortic arch, and its branches
Type III	Involvement of all the following: aortic arch, branches of the aortic arch, descending thoracic, and abdominal aorta or renal arteries
Type IV	Involvement of only the descending aorta and abdominal aorta with or without renal arteries
Type V	Combination of the types above

Angiography is considered the gold standard for vascular lesion evaluation associated with Takayasu arteritis. However, multiple noninvasive imaging modalities, including CTA, MRA, and fluorodeoxyglucose positron emission tomography ((18)F-FDG-PET) are highly sensitive and specific for detecting associated lesions even at their early stage, but it is unclear how effective it is to assess disease activity.

The most common finding in contrast to CTA and MRA is arterial mural thickening and enhancement in the arterial phase or even late phase. This can be observed in more than 90% of patients. Stenosis is another common finding that can occur anywhere from the ascending aorta to the abdominal aorta. In terms of involved branches, subclavian and common carotid arteries are more frequently affected, followed by renal arteries. Lesions involving aortic branches typically occur in the proximal segment close to branch origin. With CTA, the accuracy of measuring arterial wall thickness can be impaired due to the over-shining of intraluminal contrast [7]. This deficit can be compensated by the black blood technique used in MRA. MRA is effective in detecting the early stage of inflammatory vascular changes. Using high-resolution MRA, on a T1-weighed gadolinium-enhanced image, all three layers of the aorta can be visualized and distinguished. Maximum intensity projection sequence enables the evaluation of arterial stenosis [8].

Arterial sonography is a complementary noninvasive technique to diagnose Takayasu arteritis. On grayscale sonography, the characteristic wall thickening can be evaluated, while flow characteristics from stenosis can be evaluated on color Doppler and duplex sonography. Ultrasound is more effective in evaluating lesions in superficial large vessels such as carotid arteries or limb vessels [9].

The advantage of positron emission tomography and computed tomography (PET-CT) in diagnosing Takayasu lies in better measurement of disease activity. FDG scoring correlates well with disease activity and is more reliable for monitoring after patients are started on immunosuppression therapy [10].

In our case, the patient did not have any common presenting symptoms of Takayasu arteritis and presented with uncontrolled hypertension and atypical chest pain. There have been similar cases of initial presentation of uncontrolled hypertension in young patients who were found to have underlying renal artery stenosis due to Takayasu arteritis, which emphasizes the importance of considering it in the differential diagnosis of secondary hypertension, especially in younger females. For example, a case of a young pregnant female was reported, who presented with uncontrolled hypertension and abdominal pain and was later found to have bilateral renal artery stenosis with left subclavian stenosis due to Takayasu arteritis [5]. Another case of a 35-year-old Caucasian female was described, who presented with acute renal failure and fluid overload due to renal artery involvement secondary to Takayasu arteritis [11]. It has also been reported in one literature review that patients of Hispanic descent usually present with cardiovascular complications such as hypertension and angina as the initial manifestation of the disease [12].

## Conclusions

Takayasu arteritis can present initially as uncontrolled hypertension due to renal artery involvement in patients with no other presenting signs or symptoms of the disease. It is much more common in young women of Asian descent but can be seen in people of other ethnicities such as Hispanics, Caucasians, and Indians. A careful history and physical examination can provide clues, but imaging studies remain the cornerstone for the diagnosis. Takayasu arteritis should always be considered in the differential diagnosis for secondary hypertension, especially in the younger population, regardless of ethnicity.
